# Endocrine Autoimmunity in Pregnancy

**DOI:** 10.3389/fimmu.2022.907561

**Published:** 2022-06-29

**Authors:** Renata Świątkowska-Stodulska, Agata Berlińska, Katarzyna Stefańska, Maciej Zieliński, Sebastian Kwiatkowski, Joanna Połom, Elżbieta Andrysiak-Mamos, Piotr Wydra, Krzysztof Sworczak

**Affiliations:** ^1^ Department of Endocrinology and Internal Medicine, Faculty of Medicine, Medical University of Gdańsk, Gdańsk, Poland; ^2^ Division of Gynecology and Obstetrics, Faculty of Medicine, Medical University of Gdańsk, Gdańsk, Poland; ^3^ Department of Medical Immunology, Faculty of Medicine, Medical University of Gdańsk, Gdańsk, Poland; ^4^ Department of Obstetrics and Gynecology, Pomeranian Medical University of Szczecin, Szczecin, Poland; ^5^ Department of Internal Medicine, Connective Tissue Diseases and Geriatrics, Faculty of Medicine, Medical University of Gdańsk, Gdańsk, Poland; ^6^ Department of Endocrinology, Metabolic Diseases and Internal Diseases Pomeranian Medical University, Szczecin, Poland

**Keywords:** Hashimoto’s disease, Hashimoto’s thyroiditis, Graves’ disease, postpartum thyroiditis, autoimmune thyroid disorder, Addison’s disease, pregnancy, gestation

## Abstract

Human gestation leads to a number of physiological alterations which peak at the development of placentta known for, among many other functions, being a transient but highly potent endocrine organ. Hormonal activity of placenta is marked by its ability to continuously produce and secrete high levels of progesterone. Progesterone guards the well-being of the fetoplacental unit throughout the gestation and one of the proposed mechanisms of this principle involves the development of local and systemic immune tolerance mainly due to impediment of CD4+ lymphocyte activation. However, though these alterations are present and well-established, autoimmunity is not entirely rare and a wide spectrum of diseases can continue, or develop *de novo*, throughout the gestation or even after the delivery. Up-to-date data supports the existence of a relationship between the clinical course of chosen autoimmune diseases and levels of circulating sex steroids.

The most common autoimmune endocrinopathies in pregnant women are Hashimoto’s disease, Graves’ disease, and, more rarely, primary adrenal insufficiency in the form of Addison’s disease. Gestation can influence the clinical course of these endocrinopathies in patients who were diagnosed before conception. Multiple particles, like TSH-receptor stimulating antibodies, thyroid hormones, glucocorticoids, and anti-thyroid medications, can cross the placental barrier and evoke biological action in fetal tissues. Thyroid pathology in the form of postpartum thyroiditis is particularly prevalent in patients with positive anti-thyroperoxidase and anti-thyroglobulin antibodies. Certain populations are more at risk of developing numerous gestational complications and require regular follow-up. In our paper, we would like to address physiological, physiopathological, and clinical aspects of endocrine autoimmunity throughout human gestation, as well as special circumstances to consider in pregnant women.

## Introduction

Pregnancy is a remarkable period in a woman’s life not only owing to its psychological and social implications, but also due to vast, fast, and often deep physiological changes occurring in the short span of gestation. From a mother’s perspective, fetus can be seen as a histoincompatible transient allograft, fully able to provoke extensive immune response. To facilitate a successful pregnancy, female body must adapt and overcome the immunity targeted against the fetus. Sex steroids serve important reproductive function, but at the same time they facilitate immunomodulation crucial for the survival of the conceptus. As certain aspects of the immune system are modified, pregnancy changes the clinical course of autoimmune disorders. At the same time, autoimmune diseases, notably the endocrinopathies, can determine the course of pregnancy (1, 2). In our paper, we would like to address physiological, physiopathological, and clinical aspects of endocrine autoimmunity throughout the human gestation, as well as special circumstances to consider in pregnant women.

## Immune Adaptations in Pregnancy and the Special Role of Progesterone

Human gestation leads to a number of physiological alterations which peak at the development of placenta known for, among many other functions, being a transient but highly potent endocrine organ (3). Placenta is an independent endocrine organ that produces a number of hormones: placental growth hormone (pGH), insulin like growth factor-1 (IGF-1), human placental lactogen (HPL), growth-hormone releasing hormone (GHRH), adrenocorticotropin (ACTH), estradiol, and progesterone (P4). Pregnancy presents a complex interplay between numerous placental and non-placental hormones, most notably estrogens (E) and P4. P4 can be considered the most important for maintaining a healthy pregnancy. The immune adaptations observed during gestation affect not only the well-being of the fetus, but also the entire immune profile of the mother built around the balance between T helper type 1 (Th1) and T helper type 2 (Th2) cells. P4 and E steer the immune system *via* multiple systemic adaptations and, most likely as a coincidence, affect the clinical course of preexisting autoimmune disorders. It was observed that sex steroids favor the Th2-specific and dampen the Th1/Th17-specific response, and therefore bring exacerbation of the Th2-type disorders and amelioration of the Th1/Th17-type disorders (4, 5).

P4 can be found in steroidogenic organs, such as the adrenals, and is produced in high concentration by corpus luteum in the luteal phase of menstrual cycle. However, the abundant production of P4 by ovaries and placenta during gestation is remarkable and greatly exceeds the P4 levels seen in non-pregnant females. Over the past years, researchers proved that P4 in essential not only for preparing the uterine lining for implantation and for maintaining the proper structure of the cervix, but, most of all, for modifying the mother’s immune response to pregnancy through direct and indirect impediment of Th1 cell response and through blocking the activity and proliferation of cytotoxic T and natural killer (NK) cells (6, 7). Elevated concentration of P4 stimulates the synthesis of progesterone-induced binding factor (PIBF) (8, 9). In consequence, high levels of PIBF prompt the differentiation of CD4+ lymphocytes to Th2 cells, which produce anti-inflammatory cytokines, such as for example interleukin 4 (IL-4), interleukin 5 (IL-5), and interleukin 10 (IL-10) (8). Recent findings prove the direct effect of P4 on CD4+ T cell activation *via* altered activity of various transcription factors (7). In physiological pregnancy, a decline in Th1 action accompanies a reduction of pro-inflammatory cytokine production: interleukin 2 (IL-2), interferon γ (IFN-γ), tumor necrosis factor α (TNF-α) (8). The balance between Th1 and Th2 promotes pregnancy, leads to reduced immune response, and seems to be maintained mainly through T CD4+CD25+ cells, also known as regulatory T cells (T regs) (10). The expansion of Tregs throughout gestation proceeds due to presentation of fetal antigens to the immune cells and due to estrogen-stimulated expression of various chemokines, which is further fueled by high estrogen levels (10). After the delivery, the number of Tregs rapidly drops which allows the Th1 cells to become the dominant subgroup again, resulting in release of proinflammatory cytokines and exacerbation of autoimmune diseases. P4 hampers the humoral response *via* suppression of post-translational glycosylation of active immunoglobulins (11). Therefore, a switch in the hormonal profile of pregnant women, especially the significant rise of P4 concentration, triggers a multi-level modification of immune activity. In practice, it can be seen in pregnant women with a history of autoimmunity. It was observed that diseases such as rheumatoid arthritis, multiple sclerosis, or even systemic lupus erythematosus can remit for the span of gestation, only to flare up again after the delivery (12). Besides the above-mentioned broad adaptations there are, of course, other adjustments, such as thymic involution, modified concentrations of complement factors and regulatory proteins, and altered, at least to some extent, function of virtually all immune cell subtypes (13, 14). Nonetheless, as the main focus of this article is set on clinical aspects of endocrine autoimmunity during pregnancy, we will abstain from further deliberations.

The most common autoimmune endocrinopathies in women of reproductive age are thyroid disorders – Hashimoto’s disease and Graves’ diseases, and females seem to have a notably higher risk of developing these conditions than men (15, 16). After the delivery, post-partum thyroiditis may ensue. Primary adrenal insufficiency is certainly not as common, nonetheless it can provoke a number of complications.

Large epidemiological studies confirmed the relationship between thyroid pathologies and the occurrence of various complications threatening the well-being of mothers, fetuses, and newborns (17). Thyroid dysfunction is associated with a greater predisposition to spontaneous abortion, premature delivery, pre-eclampsia, or gestational diabetes mellitus (17–19).

The pathogenesis of autoimmune thyroid disorders is based on the activation of CD4+ lymphocytes, which can co-stimulate B lymphocytes to anti-thyroid antibody production (20). Currently, autoimmune thyroid disorders (AITDs) are believed to stem from the dysfunction of Tregs, as well as from direct cytotoxicity and cytokine-stimulated apoptosis of thyrocytes.

Clinical presentation of AITDs can be inconsistent between patients and thyroiditis can be accompanied by clinical features of either hypothyroidism or hyperthyroidism. The observed differences result from the type of recognized antigens, the presence of stimulating/blocking antithyroid antibodies, the grade of gland’s infiltration and destruction, and the degree of fibrotic changes within the thyroid.

### Autoimmune Thyroid Disorders: Euthyreosis

Not only the overt thyroid disorders can put their toll on human gestation. The mere presence of anti-thyroid antibodies, such as anti-thyroperoxidase antibodies (ATPO) and anti-thyroglobulin antibodies (ATG), can bring significant disadvantage to the mother. ATPO and ATG can be found in up to 5-14% and 3-18% of pregnant women, consecutively (1). Even euthyroid patients with positive anti-thyroid antibodies are proven to be more at risk of spontaneous abortion, premature birth, progression to hypothyroidism, and development of postpartum thyroiditis (2, 17, 21–23). The exact background of observed abnormalities is not fully clarified. One of the main theories stated that the presence of antithyroid antibodies can be seen as a sign of a wider spectrum of active autoimmunity, which may eventually provoke obstetric failures (21). This hypothesis can be further supported by the data confirming increased numbers of CD5+CD20+ B lymphocytes, known as the major triggers of autoimmunity, in women who miscarried (1). Another hypothesis focused on the elevated levels of thyrotropin (TSH) in females with positive anti-thyroid antibodies as a hint towards inadequately low levels of circulating thyroid hormones in the state of increased demand (21). The third theory connected the presence of antithyroid antibodies with reduced fertility in older females (21). The direct effect of ATPO promoting increased production of IL-2 and NK cell activation was considered as well. Recent findings proved the presence of thyroid-specific peroxidase in the endometrium and placenta which can be an additional explanation of a higher risk of miscarriage in ATPO-positive patients (24).

Despite of multiple studies demonstrating the reduction of anti-thyroid antibody titers during gestation, females suffering from autoimmune thyroiditis still display higher TSH levels than their counterparts with negative antithyroid antibodies (22). ATG and ATPO cross the placenta, however they do not provoke thyroid disorders in fetuses. Many observed changes can be explained by altered Treg activity which is increased during pregnancy and decreases after the delivery (10, 25).

As the presence of antithyroid antibodies in pregnant females increases the risk of obstetric complications, it was questioned whether early introduction of thyroid hormone supplementation in euthyroid individuals with confirmed circulating autoantibodies could be beneficial. Based on new data, it seems that low-dose levothyroxine (LT4) supplementation (50 μg daily) in euthyroid pregnant women with confirmed AITD does not benefit them in terms of infertility and spontaneous abortion (26, 27).

According to current knowledge, if female patients with a known history of autoimmune thyroid disorder choose to conceive, their thyroid function should be closely monitored (22, 28). Pregnant women with positive antithyroid antibodies should regularly monitor their TSH at least once every four weeks until the middle of gestation, and at least once around the 30th week – due to increased risk of developing hypothyroidism (29).

Positive ATPOs are a confirmed risk factor for postpartum thyroiditis and consecutive persistent hypothyroidism (postpartum thyroiditis can affect up to 30-50% patients with circulating ATPOs) (17). Therefore, thyroid function should be assessed 6-8 weeks post-delivery if ATPOs were confirmed (30).

### Autoimmune Thyroid Disorders: Hypothyroidism (Hashimoto’s Disease)

Hashimoto’s disease is the most common cause of hypothyroidism in young females, if only there is no endemic iodine deficiency. Owing to considerable increase in thyroid hormone demand during gestation (up to 50% higher than the initial needs), the risk of progression to hypothyroidism in women with positive ATPO and/or ATG is elevated even if the antibody titers drop in a spontaneous way. Following the gestational hyperestrogenism, the liver starts to produce larger amounts of thyroxine-binding globulin (TBG) and the activity of deiodinases changes (2, 23). As a result, pregnant women with autoimmune thyroid disorders can progress to subclinical or overt hypothyroidism.

Subclinical hypothyroidism (elevated TSH and free thyroxine [fT4] within the normal limits) is the most common form of thyroid pathology during gestation and it occurs in 2.5% of pregnant women. Up to 60% of women with subclinical hypothyroidism present with positive antithyroid antibodies. Overt hypothyroidism (elevated TSH, decreased fT4) is significantly less common and it occurs in 0.2-0.5% of pregnancies (31). Hypothyroidism is undoubtedly connected with numerous complications both for the mother and the fetus: spontaneous abortion, premature birth, gestational hypertension, preeclampsia, low birth weight, placental abruption, and postpartum hemorrhage (22). Untreated overt hypothyroidism in mothers results in decreased intelligence quotient in their offspring. Subclinical hypothyroidism can cause a higher risk of unfavorable events during gestation, however available data is not uniform (31, 32).

In most cases, signs and symptoms of hypothyroidism are rather scarce and can be concealed by pregnancy. The most common complaints include fatigue, drowsiness, constipation, body mass gain, cold intolerance, and dry skin. The diagnosis of hypothyroidism in pregnant females can be made based on elevated TSH and decreased fT4 concentrations (assessed for the gestational age and with reference norms set for each population of pregnant women).

According to the current guidelines, each case of overt hypothyroidism during pregnancy calls for immediate LT4 substitution. All pregnant women presenting subclinical hypothyroidism and an autoimmune thyroid disorder should be started on thyroid hormone supplementation as well; in such scenario, the diagnosis should be made based on reference norms estimated for the target population (29). Women treated for hypothyroidism before they conceived require an increase of LT4 supplementation by 30-50%, depending on the degree of thyroid damage and the time span of their disease. The target value of TSH is set between the lower reference norm of the test and 2.5 μU/ml (29, 33). Women treated with LT4 should measure their TSH once every four weeks until the midgestation and at least once near the 30th week (29).

### Autoimmune Thyroid Disorders: Hyperthyroidism (Graves’ Disease)

Graves’ disease (GD) is the most common cause of autoimmune hyperthyroidism in pregnant women (85% of cases) (2). It is caused by autoantibodies stimulating TSH receptors (TSAb). TSAbs promote the growth and activity of thyroid follicular cells, leading to thyroid hormone overproduction (20). The prevalence of hyperthyroidism resulting from GD is estimated to be around 0.2% of all pregnancies (30, 31, 34). Hyperthyroidism in pregnancy can lead to arterial hypertension, congestive heart failure, premature labor, preeclampsia, and hypermetabolic crisis within the perinatal period (35, 36). Hyperthyroidism in mothers can bring serious complications for fetuses. Besides the low birth weight, the most common unfavorable outcomes include higher chance of congenital defects, increased perinatal mortality, and, if the placenta-crossing TSAbs are elevated, fetal hyperthyroidism. Therefore, women with confirmed GD and willing to conceive should postpone the pregnancy until euthyreosis is reached (29). The risk of recurrent hyperthyroidism is significant in patients whose TSH levels are continuously decreased despite the anti-thyroid treatment, in those who received anti-thyroid drugs for shorter than 6 months, require more than 5-10 mg of thiamazole daily, demonstrate clinical signs of thyroid ophthalmopathy, present with large goiter, and/or have elevated levels of TSAbs. If the risk of recurring disease is high, anti-thyroid medication should not be stopped in the first trimester of pregnancy (29). Some of the typical signs and symptoms of hyperthyroidism can mimic those of pregnancy; the most common ones include weight loss/lack of weight gain, goiter, thyroid eye disease, heart palpitations, and loose stool (32, 37). GD-related hyperthyroidism usually accelerates in the first trimester of pregnancy, most likely due to elevated TSAb levels at the beginning of gestation. β-hCG can co-stimulate the thyroid production of hormones up to 16-18 weeks of gestation. As the pregnancy progresses and the immune system adapts, TSAb concentration drops and the clinical course of hyperthyroidism mildens. It permits a down-titration of anti-thyroid drugs, or even full discontinuation of treatment in the second half of pregnancy. Nevertheless, hyperthyroidism may recur post-partum as it happens in about 40% of GD patients (38).

Decreased TSH during pregnancy calls for total thyroxine (TT4)/fT4 and triiodothyronine testing (29, 39). The biochemical diagnosis of hyperthyroidism in pregnant women relies on the typical constellation of suppressed TSH and elevated peripheral hormones. All parameters should be assessed with special regard for the tested population. To confirm GD, TSAbs must be positive. It is crucial not only to prove the presence of TSAbs, but to measure their titer: the presence of TSAbs establishes the etiology of hyperthyroidism and their titer stratifies the risk of fetal hyperthyroidism as TSAbs cross the placental barrier. Fetal hyperthyroidism due to maternal TSAbs usually develops after the 20^th^ week of gestation. Therefore, women with TSAbs confirmed at the beginning of pregnancy should undergo consecutive testing between the 18^th^ and 22^nd^ week of gestation (to stratify the risk of fetal hyperthyroidism) and between the 30^th^ and 34^th^ week (to assess the risk of neonatal hyperthyroidism).

Subclinical hyperthyroidism does not call for immediate start of antithyroid drugs. However, many women suffering from GD eventually develop overt hyperthyroidism and might require appropriate medications. Propylthiouracil (PTU) is the drug of choice in the first trimester of pregnancy (29, 32). When fetal organogenesis is finished (16^th^ week of gestation), PTU can be replaced by thiamazole. Treatment should be aimed at the lowest effective dose of anti-thyroid drugs possible, with target TT4/fT4 set at the upper limit of the laboratory norm for gravid women. At first, thyroid function in hyperthyroid pregnant women should be assessed once every 2-4 weeks, and later on, as the target hormone levels are reached, it can be checked once every 4-6 weeks. Antithyroid treatment can be entirely stopped in about 20-30% of patients once they reach the third trimester of pregnancy and/or fall into remission (29).

### Postpartum Thyroiditis

Postpartum thyroiditis (PPT) is characterized by transient abnormalities in thyroid function starting within the first year after delivery, spontaneous abortion, or induced abortion. PPT usually occurs in women with a pre-existing autoimmune thyroid disorder. PPT can affect up to 33-50% of women with positive ATPO (36). PPT can arise in either euthyroid patients who were never substituted with LT4 or hypothyroid patients receiving LT4 due to subclinical or overt hypothyroidism, however, it is ten times more likely to be diagnosed in females who were euthyroid before conception (36). More so, women who were euthyroid at the beginning of their pregnancy, regardless of the LT4 supplementation status, have a four-fold higher chance of developing PPT as compared with their counterparts whose TSH levels exceeded the normal limits (36).

PPT is a destructive inflammatory process leading to uncontrolled release of thyroid hormones from damaged cells. Usually, the clinical course of PPT is triphasic: initial hyperthyroidism is followed by hypothyroidism, and, eventually, euthyroidism. Sometimes, the thyroid dysfunction can have only two phases: hyperthyroidism or hypothyroidism with subsequent euthyroidism.

The hyperthyroid phase tends to manifest between 2 and 6 months postpartum. It can be entirely asymptomatic or the signs and symptoms, such as fatigue, irritability, heart palpitations, and weight loss, are mild. The hypothyroid phase develops between 3 and 13 months after the delivery and is usually clinically overt with signs and symptoms including dry skin, cold intolerance, and loss of concentration. PPT turns into permanent hypothyroidism in 10-20% of cases.

The hyperthyroid phase of PPT must be differentiated from recurring hyperthyroidism in the course of GD. The hyperthyroid phase of PPT does not require treatment with anti-thyroid drugs, and β-antagonists are effective in relieving the symptoms. TSH should be checked once every 6-8 weeks to spot the beginning of the hypothyroid phase. In symptomatic cases of hypothyroidism, LT4 supplementation should be considered and thyroid function should be assessed every 4-8 weeks until the stabilization is reached. Clinical implications for continuous LT4 use should be reevaluated after 12 weeks of treatment.

### Autoimmune Primary Adrenal Insufficiency (Addison’s Disease)

Autoimmune primary adrenal insufficiency, also known as Addison’s disease (AD), usually targets females of reproductive age. The incidence of AD in pregnant women seems to be on the rise. According to a cohort study of 7.7 million births in the US, the initial incidence of AD in pregnant women was approximately 5.5/100,000 and it raised up to 9.6/100,000 over the next 9 years (40). Properly treated AD does not affect fertility; however, it is often accompanied by other autoimmune disorders such as type 1 diabetes or autoimmune thyroiditis which can take their toll on the mother’s health, fertility, and on the pregnancy outcomes (41, 42). AD carries an increased risk for the mother as it may result in a potentially deadly adrenal crisis. In addition, analysis of perinatal complications in pregnant AD patients compared with their healthy counterparts proved a higher incidence of premature birth, cesarean section, impaired wound healing, infections, thromboembolic events, blood transfusions, and prolonged hospitalizations in AD (40).

AD typically progresses slowly over years and its onset is insidious. Positive anti-21-hydroxylase antibodies can be viewed as an early marker of developing AD as they can be tracked in the patient’s serum even before the first signs and symptoms of the disease appear. The autoimmunity in AD stems from autoreactive CD8+ T lymphocytes targeting 21-hydroxylase’s peptides (43).

It is rare for AD to first manifest during pregnancy; usually, the disease is diagnosed earlier and proper management of AD actually allows conception. New diagnosis of AD during gestation is very difficult as the signs and symptoms of the disease often overlap with those of an otherwise healthy pregnancy. The typical clinical manifestations include nausea, vomiting, syncope (due to hypotonia), hyperpigmentation of nipples, areolas, and skin. A subset of signs and symptoms particularly characteristic for low glucocorticoid levels are inadequate body mass gain related to pregnancy, pain in the abdomen, orthostatic hypotension, tachycardia, hyponatremia, hypoglycemia, lymphocytosis, and eosinophilia. Physiological pregnancy leads to an increase in circulating cortisol and ACTH, making the typical endocrine diagnostics problematic. Morning cortisol below 3 μg/dl confirms the diagnosis of AD, and cortisol above 19 μg/dl – excludes it. In ambiguous cases, the initial screening can be followed by synthetic ACTH stimulation test which is routinely used in general population (44).

AD requires urgent and continuous supplementation of glucocorticoids and mineralocorticoids. Hydrocortisone is the glucocorticoid of choice as it does not pass through the placenta and does not suppress fetal adrenal glands. In the first and second trimester, the substitution of hydrocortisone can usually be maintained at the same level (15-30 mg daily, divided into two or three doses). After the 24^th^ week of gestation, the doses are often readjusted and increased by 20-40% due to physiological hypercortisolemia present in healthy pregnant women (45). Fludrocortisone, a synthetic mineralocorticoid, can be used the same way as in the general population – 0.05-0.2 mg daily, with close monitoring of blood pressure and serum electrolytes. Both vaginal birth and cesarean section in AD patients call for an increased glucocorticoid supplementation. 100 mg of intravenous hydrocortisone should be administered without any delays during delivery, and if there is a need for cesarean section, additional 100 mg i.v. every 6 hours should be introduced. Increased hydrocortisone demand is usually present up to 2 days post-delivery; later on, the doses can be titrated back to the normal maintenance dose (41, 46).

## Summary

Autoimmune thyroid disorders have a high prevalence in women of reproductive age and can become an important clinical dilemma during gestation. Addison’s disease is definitely less common but, if unrecognized and untreated, it can be dangerous for pregnant patients. Gestation provokes numerous immunological and hormonal adaptations, and pregnant women with a history of autoimmune endocrine disorders require specialist care. Proper follow-up and treatment reduce the risk of unfavorable outcomes, such as for instance miscarriage, premature birth, low birth weight, or preeclampsia.

## Author Contributions

RŚ-S, AB: These authors contributed equally to this work and share first authorship, conceived the idea of the work, contributed to the design of publication and reference collection, and were responsible for preparing the manuscript. KaS, MZ, SK, JP, EA-M: Involved in preparing the manuscript. KS: proof-reading and revision of the manuscript. All authors contributed to the article and approved the submitted version.

## Conflict of Interest

The authors declare that the research was conducted in the absence of any commercial or financial relationships that could be construed as a potential conflict of interest.

## Publisher’s Note

All claims expressed in this article are solely those of the authors and do not necessarily represent those of their affiliated organizations, or those of the publisher, the editors and the reviewers. Any product that may be evaluated in this article, or claim that may be made by its manufacturer, is not guaranteed or endorsed by the publisher.
